# Tactile direction discrimination in humans after stroke

**DOI:** 10.1093/braincomms/fcaa088

**Published:** 2020-06-30

**Authors:** Linda C Lundblad, Håkan Olausson, Pontus Wasling, Katarina Jood, Anna Wysocka, J Paul Hamilton, Sarah McIntyre, Helena Backlund Wasling

**Affiliations:** f1 Department of Clinical Neurophysiology, Sahlgrenska University Hospital, S-413 45 Gothenburg, Sweden; f2 Institute of Neuroscience and Physiology, University of Gothenburg, S-405 30 Gothenburg, Sweden; f3 Department of Biomedical and Clinical Sciences, Center for Social and Affective Neuroscience, Linköping University, SE-581 83 Linköping, Sweden; f4 Department of Neurology, Sahlgrenska University Hospital, S-413 45 Gothenburg, Sweden

**Keywords:** touch, direction discrimination, somatosensory system, stroke, structural MRI

## Abstract

Sensing movements across the skin surface is a complex task for the tactile sensory system, relying on sophisticated cortical processing. Functional MRI has shown that judgements of the direction of tactile stimuli moving across the skin are processed in distributed cortical areas in healthy humans. To further study which brain areas are important for tactile direction discrimination, we performed a lesion study, examining a group of patients with first-time stroke. We measured tactile direction discrimination in 44 patients, bilaterally on the dorsum of the hands and feet, within 2 weeks (acute), and again in 28 patients 3 months after stroke. The 3-month follow-up also included a structural MRI scan for lesion delineation. Fifty-nine healthy participants were examined for normative direction discrimination values. We found abnormal tactile direction discrimination in 29/44 patients in the acute phase, and in 21/28 3 months after stroke. Lesions that included the opercular parietal area 1 of the secondary somatosensory cortex, the dorsolateral prefrontal cortex or the insular cortex were always associated with abnormal tactile direction discrimination, consistent with previous functional MRI results. Abnormal tactile direction discrimination was also present with lesions including white matter and subcortical regions. We have thus delineated cortical, subcortical and white matter areas important for tactile direction discrimination function. The findings also suggest that tactile dysfunction is common following stroke.

## Teaser

We examined a group of patients with first time stroke to study which brain regions are important for tactile direction discrimination. We found impairment associated with lesions in previously identified somatosensory, insular, and prefrontal cortical areas. In addition, we found impairment associated with lesions in subcortical and white matter areas.

## Graphical Abstract

**Graphical Abstract:**
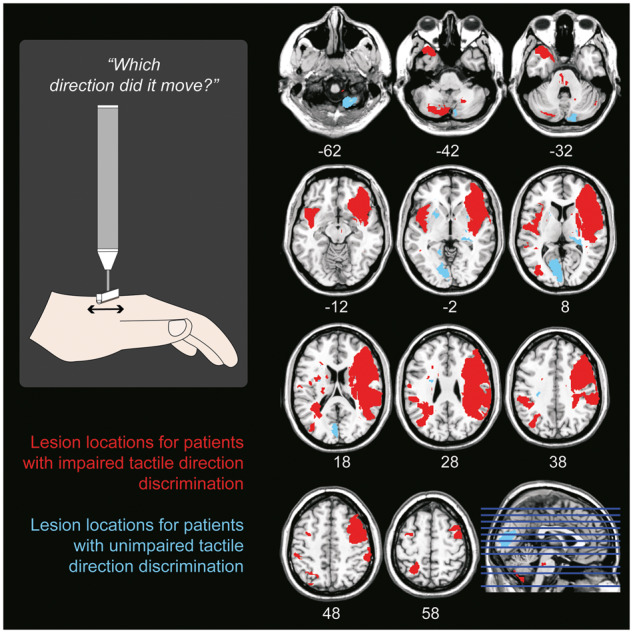

## Introduction

Humans have a sophisticated sensitivity to the features of movements across the skin, which relies on complex cortical processing (Pei *et al.*, 2010; McIntyre *et al.*, 2016). Tactile direction discrimination (TDD) is the ability to tell the direction of an object’s movement across the skin, and disturbances in TDD reveal neurological dysfunction (Tshlenow, 1928; Olausson *et al.*, 1997; Löken *et al.*, 2010). However, the importance of tactile sensation for daily function may still be underestimated in many clinical settings (Birznieks *et al.*, 2012). A better understanding of the cortical regions involved in processing tactile motion would make TDD testing more informative and widen its scope. To this end, we investigated, for the first time, how lesions of different brain areas affect TDD.

There are two important cues to direction of a tactile stimulus that moves tangentially to the skin surface. The first is skin stretch, produced by high friction stimuli, such as tangential skin pull (Edin, 1992; Olausson *et al.*, 1998). The second cue comes from the stimulation of successive positions on the skin along the path of motion (sometimes called the spatiotemporal cue). Tactile direction judgements are typically more sensitive in the presence of skin stretch (Olausson *et al.*, 1998; Gleeson *et al.*, 2010), likely due to activity in the slowly adapting type 2 peripheral afferents, which are highly sensitive to the direction of skin stretch (Olausson *et al.*, 1998, 2000). Low friction stimuli, such as a rolling wheel, moving air jet or a tactile array, cause little to no skin stretch, providing only the successive positions cue for movement direction (Norrsell and Olausson, 1994; McIntyre *et al.*, 2016). Such stimuli engage all classes of peripheral low-threshold mechanoreceptor afferents, both myelinated and unmyelinated, and the directional information is most likely conveyed via central integration of the successively activated primary afferents with neighbouring receptive fields (Gardner and Palmer, 1989; Srinivasan *et al.*, 1990; Pei *et al.*, 2010; McIntyre *et al.*, 2016; Saal *et al.*, 2017).

In most natural touch stimuli, both skin stretch and successive positions cues are present, and contribute simultaneously to perceived direction of movement (Seizova-Cajic *et al.*, 2014). Both cues are also sensitive to disturbances in the dorsal column pathways (Bender *et al.*, 1982; Hankey and Edis, 1989). Using functional magnetic resonance imaging (fMRI) with healthy humans, we have shown that direction discrimination of tactile motion applied to the leg is processed in a wide set of cortical regions, and that the specific areas engaged depend on the cues present in the stimulus. Specifically, TDD of motion including skin stretch is processed in the opercular parietal area 1 of the secondary somatosensory cortex (S2 OP1), the dorsolateral prefrontal cortex (DLPFC) and the anterior insular cortex (Backlund Wasling *et al.*, 2008; Lundblad *et al.*, 2010). TDD of motion with only the successive positions cue is processed in the same areas as that with skin stretch and in addition, in primary somatosensory cortex (S1) and posterior insular cortex (IC) (Lundblad *et al.*, 2011). Individual neurons in primate S1 also show direction-selective responses to stimuli that provide only the successive positions cue (Pei *et al.*, 2010).

In the present study, we investigated the capacity for TDD of motion on the foot and the hand in a group of patients with first-time stroke. We have previously presented a clinical method for quantitative testing of TDD (Olausson *et al.*, 1997; Löken *et al.*, 2010). The stimulus was a hand-held probe that the experimenter moved across the skin. It generated both skin stretch and successive position cues. In previous fMRI studies of healthy individuals, we selectively studied the skin stretch input and successive positions input. But as both cues contribute simultaneously to direction perception, rather than simply providing redundancy (Seizova-Cajic *et al.*, 2014), our strategy here was to provide rich peripheral input in order to engage the full set of relevant cortical regions. The aim of the current study was to identify brain regions crucial for judging the direction of tactile motion as measured by a TDD task. We hypothesized that a stroke that affects the areas previously identified by fMRI as being engaged during a TDD task (S1, S2 OP1, DLPFC, anterior insular cortex, posterior IC) would be associated with a disturbance in TDD.

## Materials and methods

We tested TDD on first-time stroke patients’ hands and feet within 2 weeks (‘acute’ stage) and at 3 months after the stroke. At the visit 3 months after the stroke, the patients also underwent a structural MRI scan to delineate the lesions. We also tested TDD on a healthy control group, whose scores provided baseline values. The experiments were undertaken with the understanding and written consent of each participant. The study was performed according to the Declaration of Helsinki after approval of the Ethics Committee of University of Gothenburg.

### Patients

Forty-four previously neurologically healthy patients with first-time stroke (age 27–82 years, 32 men, 12 women) were recruited for the study. The sample size was similar to a study on the effects of diabetes mellitus on TDD (Löken *et al.*, 2010). The patients were recruited between 2005 and 2011 from the Stroke Units at the Sahlgrenska University Hospital, Kungälv Hospital, Södra Älvsborg Hospital and Skaraborg Hospital, Sweden. Patients were considered for the study only when both a medical doctor associated with the study and the TDD examiner were present. Exclusion criteria were previous neurological disease including polyneuropathy, inability to speak, symptoms of extinction or inability to understand and follow instructions. Both visual and sensory extinction were evaluated and in cases where these were present, the patients were excluded from the study. To test tactile extinction, the examiner touched the left or right hand or both hands of the patient simultaneously. The patient kept their eyes closed during the test and indicated, verbally or by pointing, which hand was touched. To test visual neglect, the examiner held up both their hands in the patient’s temporal visual field and moved their fingers on the right hand, the left hand or both hands simultaneously. The patient kept their eyes open and fixated on the examiner’s nose during the test and indicated, verbally or by pointing, which hand’s fingers were moving. Each version of the test was repeated three times (left side, right side and simultaneous stimulation). If a patient consistently reported sensing or seeing only the stimulus on the ipsilesional side when both sides were stimulated, the patient was considered to display extinction and thus impaired attention and was excluded from the study. There was no evaluation of hemispatial neglect independently of extinction. Even though extinction and neglect often co-occur, it is debated whether extinction should be considered a ‘weak form of neglect’ or a separate phenomenon (Bonato, 2012).

All patients had been diagnosed using MR or CT scan in combination with clinical examination and history. They were diagnosed with stroke due to focal ischaemia (*n* = 38) or intracerebral haemorrhage (*n* = 6). The causes of stroke were cryptogenic (*n* = 16), lacunar (*n* = 11), vascular dissection (*n* = 5), cardiac embolism (*n* = 7), occluded internal carotid artery (*n* = 3), antiphospholipid syndrome (*n* = 1) and migraine (*n* = 1); 72.7% (*n* = 32) of the patients had hypertension and 20.5% (*n* = 9) were untreated at admission. Atrial fibrillation was found in 18.2% (*n* = 8), hyperlipidaemia in 36.4% (*n* = 16) and 20.5% (*n* = 9) were smokers. Intravenous thrombolysis was performed in four patients and thrombectomy in three. All the patients, except five for whom we lack data, were right-handed. Six patients underwent neuropsychological evaluation during clinical care, but with different test batteries. Therefore, the results of these tests were not included in the study.

Three months after stroke, the study group consisted of 28 remaining patients (age 29–82, mean age 57, 20 men), after 16 were excluded for the following reasons: declined further participation (*n* = 7), second stroke (*n* = 1), no detectable lesion on the MRI (*n* = 3), claustrophobia in the MRI (*n* = 3) or technical problems during the scanning or TDD examination (*n* = 2). Out of the six original patients with haemorrhage, only two remained in the study after 3 months. We included these two patients because haemorrhage was not initially an exclusion criterion, and both patients had relatively small bleedings that were resorbed after 3 months (see Table 1).

**Table 1:** Patient characteristics for those who were present for the 3-month follow-up

Patient	Age	Gender	Type of stroke	Rehabilitation
1	65	M	Infarction	No rehabilitation
2	50	M	Infarction	No rehabilitation
3	52	M	Infarction	Inpatient rehabilitation (7 days)
4	55	F	Haemorrhage	Inpatient rehabilitation (9 days)
5	59	M	Infarction	Inpatient rehabilitation (9 days)
6	76	F	Infarction	No rehabilitation
7	50	M	Infarction	Outpatient rehabilitation
8	62	F	Infarction	Inpatient rehabilitation (100 days)
9	64	M	Infarction	No rehabilitation
10	73	F	Infarction	No rehabilitation
11	46	F	Infarction	Outpatient rehabilitation
12	47	M	Infarction	Outpatient rehabilitation
13	50	M	Infarction	Inpatient rehabilitation (29 days)
14	58	F	Infarction	Inpatient rehabilitation (16 days)
15	58	M	Infarction	Outpatient rehabilitation
16	60	M	Infarction	Outpatient rehabilitation
17	63	M	Infarction	No rehabilitation
18	65	M	Infarction	Outpatient rehabilitation
19	68	M	Infarction	No rehabilitation
20	82	F	Infarction	No rehabilitation
21	73	M	Infarction	No rehabilitation
22	29	M	Infarction	Inpatient Rehabilitation (19 days)
23	41	F	Infarction	No rehabilitation
24	42	M	Infarction	Outpatient rehabilitation
25	42	M	Infarction	No rehabilitation
26	53	M	Haemorrhage	Inpatient rehabilitation (29 days)
27	59	M	Infarction	Outpatient rehabilitation
28	66	M	Infarction	Outpatient rehabilitation

F: female; M: male.

### Normative data

Fifty-nine healthy participants (age 22–68 years, mean age 43 years, 25 men and 34 women) provided baseline TDD scores, the normative data against which the patients were compared. TDD was measured on the dorsum of the left hand in 34 healthy participants (age 22–68 years, mean age 49 years, 18 men and 16 women), and on the dorsum of the left foot in 43 healthy participants (age 22–68 years, mean age 44 years, 15 men and 28 women). To ensure that the participants over 55 years of age had normal conduction velocities in the sensory nerves innervating the skin areas being examined, these participants underwent a nerve conduction examination. Sensory conduction velocity, amplitude, latency and duration were examined in the ulnar, radial and the peroneal superficial nerves by electrical surface stimulation (a standard clinical technique). Inter-examiner reliability was studied between two examiners who performed TDD measurements on 16 of the healthy participants (age 30–68 years, mean age 54 years, 8 men and 8 women). Each participant was examined twice on the same day by two different examiners.

### Tactile direction discrimination

**Figure 1:**
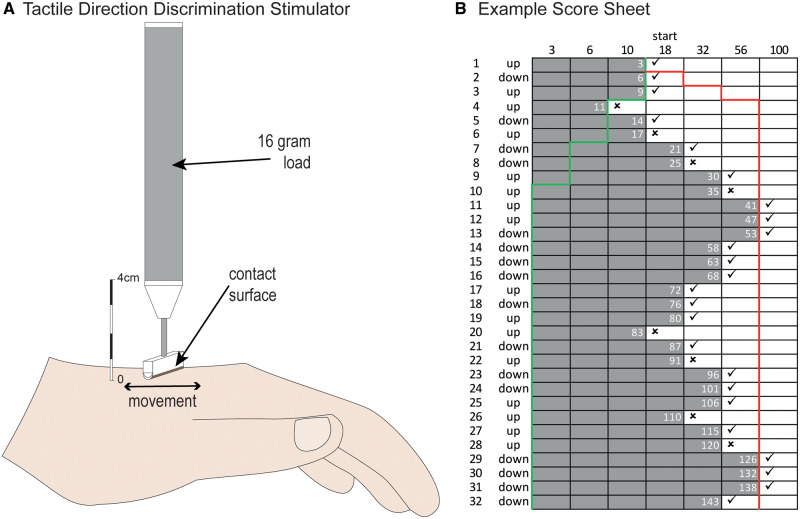
**The TDD test.** (**A**) Hand-held stimulator used for the TDD test. The contact surface consisting of a half cylinder (diameter 4 mm, length 15 mm) is covered by fine woven fabric (Leucoplast, Hamburg, Germany) to make a frictional stimulation when it is moved across the skin. (**B**) Example scoring sheet for the TDD test. Stimulation distances were selected from a logarithmic series (3, 6, 10, 18, 32, 56 or 100 mm) (Olausson *et al.*, 1997). The test started with a single motion stimulus applied over a distance of 18 mm, and later stimulation distances were selected depending on the participant’s performance. If the participant gave an incorrect response, the following stimulation distance was increased to the next longer distance in the series (e.g. from 18 to 32 mm). Alternatively, if the participant answered correctly three times in a row, the stimulation distance was decreased to the next shorter distance in the series (e.g. from 18 to 10 mm). The same procedure was continued for 32 trials with an equal number of stimulations in both directions (in a pseudorandomized order). The score is the number of boxes to the left of the marked responses, which approximates the area under the curve (highlighted in grey, cumulative score shown in white figures). If the participant continuously gave correct answers, the shortest stimulation distance (3 mm) would be delivered until 32 trials were completed (shown as a green trace branching off to the left), producing a minimum possible score of 18. If the participant failed to give correct answers, the longest stimulation distance would be delivered until 32 trials were completed and the incorrect answers would be noted (shown as a red trace branching off to the right), producing a maximum score of 186 (Löken *et al.*, 2010).

The testing procedure and equipment are the same as in our previous studies (Norrsell *et al.*, 2001). We used a hand-held stimulator (Fig. 1A) with a contact surface consisting of a half cylinder (diameter 4 mm, length 15 mm) covered by fine woven fabric (Leucoplast, Hamburg, Germany). Testing was made bilaterally on the dorsum of the hands and feet with a vertical load of 16 g. A forced-choice method was used, and the stimulator was moved at 10 mm/s over a predetermined distance in either proximodistal or distoproximal direction in a pseudo-random order (Durup, 1967). The stimulation area (100 mm) was marked with parallel lines at 3 mm intervals with a rubber stamp, and the stimuli were applied to locations distributed pseudo randomly within the marked area. The participants had their eyes closed and verbally reported the direction of the movement (‘down’ for proximodistal or ‘up’ for distoproximal). Stimulation distances were selected from an approximately logarithmic series (3, 6, 10, 18, 32, 56 or 100 mm) and followed an adaptive protocol, getting easier (longer distances) if the participant answered incorrectly, and more difficult (shorter distances) after three correct responses (Olausson *et al.*, 1997). The answers were marked in a scoring sheet to provide a TDD score that approximated the area under the curve, expressing the capacity of the participants to discriminate direction of tactile motion (Fig. 1B).

### Structural MRI

Three months after the stroke, the patients went through a structural MRI scan using a 1.5-T Philips Intera scanner (Eindhoven, Netherlands) with a standard head coil, following the hospitals’ clinical routine protocols. The anatomical scans were acquired with 2 mm thick slices using a high-resolution T2-weighted anatomical protocol (TR, 5000 ms; TE, 11 ms). The lesions were masked manually with the help of an experienced neuroradiologist using MRICron software (2009; http://people.cas.sc.edu/rorden/mricron/install.html, accessed 13 July 2020). The mask was then normalized into a three-dimensional coordinate system of the human brain (Montreal Neurological Institute and Hospital, MNI, space) using Statistical Parametric Mapping (SPM8, http://www.fil.ion.ucl.ac.uk/spm/, accessed 13 July 2020). The brain regions were defined anatomically from the structural MR image by the neuroradiologist. The target regions were defined as in previous fMRI studies using PickAtlas (Maldjian *et al.*, 2003). For subdivision of S2 into OP1–4, we used stereotaxic maps defined in MNI coordinates (Eickhoff *et al.,* 2006).

### Statistical analysis

For clinical applications, it is useful to be able to categorize a TDD score as impaired or normal. To do this, previous studies used a simple cut-off based on the TDD scores of a healthy sample (Norrsell *et al.*, 2001; Löken *et al.*, 2009). However, there is some evidence that TDD capacity declines with age (Olausson *et al.*, 1997). We tested for a linear effect of age on TDD score using linear regression, and where appropriate, adjusted for the effects of age. We similarly tested for any effect of sex. The cut-off was set as the 90th percentile of the normative sample.

To test an alternative for deriving the threshold, we also performed individual deficit analysis. For the two hands, we tested whether each patient’s scores were significantly different from the normative sample (Singlims_ES.exe, Crawford *et al.*, 2010). Because the scores obtained for the feet varied with age, we tested whether each patient’s scores were significantly different from the regression line predicted by age in the normative sample (regdiscl.exe, Crawford and Garthwaite, 2006). Using a cut-off for raw TDD scores that were worse than 90% of healthy controls according to the Crawford tests, we compared this to our strategy described above using the 90th percentile for each of 10 age groups. The two strategies resulted in very similar results—although if using the Crawford test criteria, one patient (no. 21) no longer qualified for impairment at the 3-month follow-up. The 90th percentile approach was retained and used for further analyses.

To test the hypothesis that a stroke affecting the areas previously identified by fMRI as being engaged during a TDD task would be associated with a disturbance in TDD, we divided the stroke patients into two groups: those with lesions in any of the target areas (S1, S2 OP1, DLPFC, anterior insular cortex, posterior IC) and those with lesions in other areas. We then performed a Fisher’s exact test to compare the incidence of TDD impairment in these two groups. The sample size and heterogeneity of lesions in our sample did not support separately testing the effects of lesions to each of the target areas.

### Node-level symptom mapping

For exploratory analysis, we calculated relative TDD scores = (raw TDD score − threshold)/threshold, using the 90th percentile cut-off described above. We had planned to perform voxel-level symptom mapping (Meyer *et al.*, 2016), but the lesion locations that we observed were highly variable, and as a result, we were unable to perform meaningful statistics using voxel-level symptom mapping. For any given voxel, there were at most four patients with lesions at that voxel location, and the largest contiguous cluster consisted of only six voxels.

Therefore, to assess whether there were significant relationships between lesion location and functional impairment in a data-driven way, we conducted node-level symptom mapping as an alternative to voxel-level symptom mapping. This approach has the benefit of increased sensitivity due to fewer statistical tests run and the corresponding reduced penalty for family-wise error correction. Further, this approach automatically situates findings in the context of independently defined functional nodes of the brain. For our implementation, we used a resting fMRI parcellation from Yeo *et al.* (2011). Specifically, we broke the seven-network, liberal parcellation from Yeo *et al.* into 43 distinct, contiguous sub-regions or nodes, and determined the TDD capacity associated with the intersection between lesions and functional nodes. Inclusion in the node-level symptom mapping analysis was subject to the following conditions: (i) only nodes with more than 100 voxels were considered; (ii) a patient counted as having a lesion in that node only when the lesion covered either at least 100 voxels within the node, or 10% of node size, whichever was smaller (as nodes vary a lot in size); (iii) only nodes with at least four patients who had sufficient lesion in the node according to criterion two were analysed. One node, Node 4, qualified for analysis. It is a large node covering most of the primary motor and sensory cortices (Supplementary Fig. 1).

### Data availability

TDD data and analysis scripts are available on a public repository (McIntyre, 2020). The remaining data are available on request from the corresponding author, and are not publicly available due to their containing information that could compromise the privacy of research participants.

## Results

### Tactile direction discrimination

**Figure 2:**
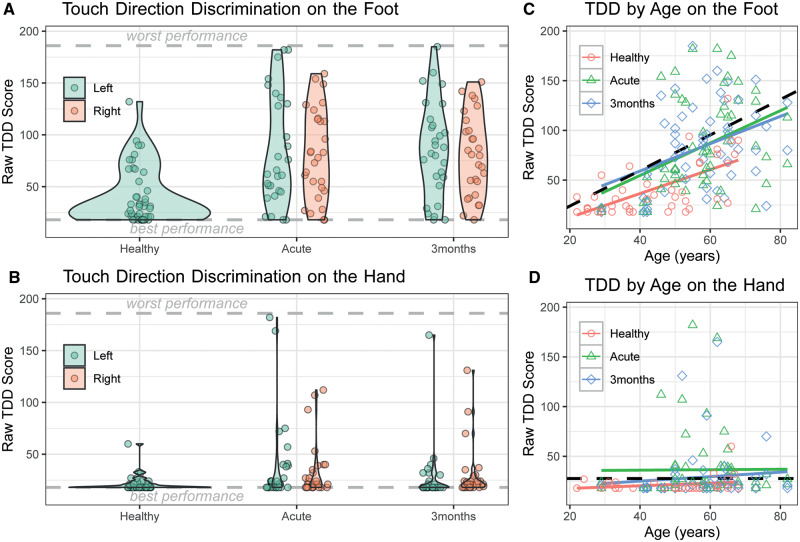
**Raw TDD scores.** Data are shown for the 28 patients who participated in the 3-month follow-up, and the healthy controls who provided baseline scores for the foot (*n* = 34) and the hand (*n* = 43). [**A** (foot) and **B** (hand)] The distribution of scores for the different tests performed. Data points are jittered on the *x*-axis to prevent over-plotting. [**C** (foot) and **D** (hand)] The relationship between TDD and age. The dashed lines show the threshold we used for determining abnormal TDD scores.

In the healthy group, the mean raw TDD score on the foot was 41.1 (SD = 27.4), and on the hand, it was 21.5 (SD = 7.9), indicating that participants had better TDD on the hand than on the foot (Fig. 2A and B). There was no significant difference in TDD measured by the two examiners, for either the hand, *P* = 0.289 or the foot, *P* = 0.778 (Student’s *t*-test). Correlation between the two examiners for TDD on the left hand (*R*^2^ = 0.9276) and on the left foot (*R*^2^ = 0.3916) can be seen in Supplementary Fig. 2.

In order to establish a threshold score for impairment, we first checked for sex and age covariates. Since the results from testing on the hands were not normally distributed (Fig. 2B), we calculated the 90th percentile in age groups of 10 years (20–29, 30–39, 40–49, 50–59 and 60–69). There were no significant differences in raw TDD scores between men and women and consequently, the data were pooled (Mann–Whitney *U* test, hand, *P* = 0.144, foot, *P* = 0.117). Raw TDD scores on the foot varied significantly with age [*F*(1,41) = 30.0, *P* = 2.41e−06], with each additional year being associated with a TDD score 1.2 points worse. For this reason, the threshold for the foot depended on age (threshold = 1.786 × age − 11.77, Fig. 2C). For the hand, there was no significant effect of age [*F*(1,32) = 1.9, *P* = 0.181], so we used a flat threshold (threshold = 27.7, Fig. 2D).

### Acute phase

Forty-four patients (age 27–82, mean age 59, 32 men) were examined in the acute phase (within 2 weeks from the stroke). Twenty-nine patients (aged 27–82 years, mean age 63, 19 men) had abnormal TDD for at least one of the four sites tested, and the remaining 15 had normal TDD (age 29–72 years, mean age 51, 13 men) for all sites.

### Three months after stroke

**Figure 3:**
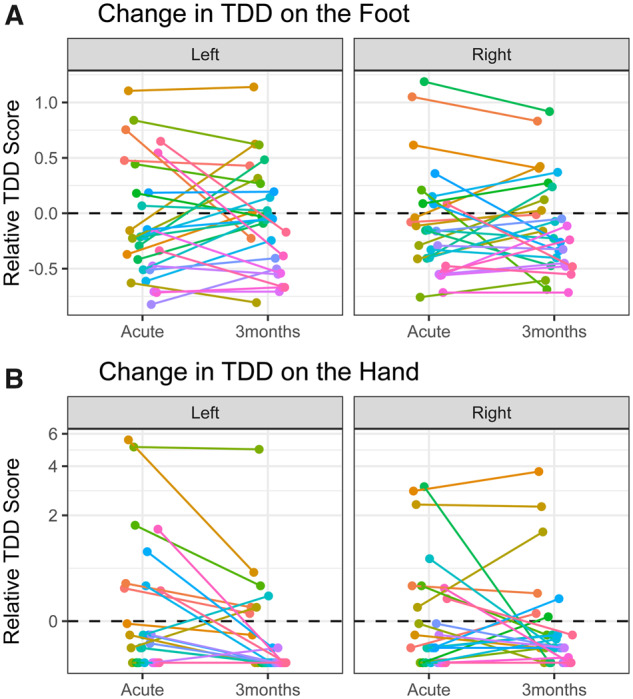
**Change in TDD scores from acute testing to the 3-month follow-up (*n* = 28).** (**A**) Data for the foot. (**B**) Data for the hand. Different coloured lines connect the scores for each individual at the two time-points. Data points are jittered on the *x*-axis to prevent over-plotting.

Considering only the 28 patients who participated in the 3-month follow-up, 21 had abnormal TDD for at least one tested site (Table 2). Comparing the TDD scores (relative to the impairment threshold) in the acute phase compared to 3 months after the stroke (pooling left and right scores), we found no significant difference when testing on the feet [mean difference in relative TDD score = −0.01, *t*(55) = 0.16, *P* = 0.971, paired samples *t*-test]. However, on the hands, TDD improved from the acute phase to 3 months after the stroke [mean difference in relative TDD score = 0.27, *t*(55) = 2.201, *P* = 0.032, paired samples *t*-test]. These data are shown in Fig. 3.

**Table 2:** Patient characteristics and TDD results

TDD acute phase					TDD 3 months after stroke				
Patient	R Hand	L Hand	R Foot	L Foot	R Hand	L Hand	R Foot	L Foot	Lesion
1	−0.24	0.41	−0.06	0.50	0.08	0.08	0.00	0.45	Right WM
2	0.44	0.48	1.09	0.78	0.34	0.16	0.86	−0.21	Left **IC**
3	2.86	−0.03	0.64	−0.36	3.73	−0.13	0.43	0.03	Bilateral WM, BS, cerebellum
4	−0.13	5.57	−0.02	1.14	−0.24	0.66	0.45	1.18	Right **IC**, LN, internal capsule
5	2.36	−0.13	−0.10	−0.14	2.29	−0.35	0.04	0.65	Left occipital and frontal lobule, bilateral parietal
6	0.16	−0.24	−0.40	−0.62	1.53	0.16	−0.15^a^	−0.80	Right thalamus, cerebellum, WM
7	−0.03	−0.35	−0.28	−0.21	−0.35	−0.35	0.14	0.34	Right medulla oblongata
8	−0.35	5.10	−0.75	0.87	−0.24	4.96	−0.60	0.64	Right **IC, DLPFC, S2 OP1**
9	0.44	1.71	0.23	0.47	−0.13	0.44	−0.68	0.29	Right thalamus, WM
10	−0.35	−0.35	0.11	0.20	0.05	−0.35	0.29	−0.01	Right CN, putamen
11	3.04	−0.35	1.23	−0.41	−0.35	−0.35	0.95	−0.08	Left medulla oblongata
12	−0.35	−0.35	−0.14	−0.28	−0.35	−0.35	−0.46	0.51	Left **S2 OP1**, cerebellum
13	−0.24	−0.24	−0.40	−0.20	−0.13	−0.35	0.26	−0.04	Left BS (Pons)
14	−0.24	−0.24	−0.14	0.09	−0.24	−0.35	−0.21	0.04	Right **IC**, WM
15	0.91	−0.13	−0.39	−0.50	−0.24	0.30	−0.06	−0.00	Bilateral WM
16	−0.35	−0.35	−0.32	−0.19	−0.17	−0.35	−0.39	0.16	Right WM
17	−0.24	0.44	0.17	−0.61	−0.13	−0.35	0.39	−0.23	Left thalamus
18	−0.24	1.06	0.10	−0.13	0.26	−0.24	−0.33	−0.04	Right **IC**, **DLPFC**
19	−0.24	−0.13	0.38	0.20	−0.24	−0.35	−0.25	0.21	Right **IC, S2 OP1**
20	−0.03	−0.21	−0.15^a^	−0.50	−0.24	−0.35	−0.03^a^	−0.40	Right BS (Pons), bilateral **IC**, WM
21	−0.35	−0.13	−0.28^a^	−0.82	−0.35	−0.35	−0.31^a^	−0.49	Left **S2 OP1**, bilateral WM
22	−0.13	−0.35	−0.54	−0.47	−0.24	−0.24	−0.44	−0.54	Right BS
23	−0.35	−0.35	−0.55	−0.70	−0.35	−0.35	−0.47	−0.70	Left WM
24	−0.35	−0.35	−0.55	−0.15	−0.35	−0.35	−0.10	−0.53	Bilateral cerebellum
25	−0.35	−0.35	−0.71	−0.71	−0.31	−0.35	−0.71	−0.66	Right cerebellum
26	0.41	1.60	−0.52	0.57	−0.35	−0.35	−0.23	−0.37	Bilateral putamen, WM
27	−0.35	−0.35	−0.47	−0.33	−0.35	−0.35	−0.54	−0.66	Left occipital lobule
28	0.26	0.37	0.10	0.68	−0.13	−0.35	−0.47	−0.16	Left LN, CN, right putamen, internal capsule

The patients are presented in the order of the number of tested areas with abnormal TDD 3 months after the stroke. The values are the relative TDD score, which indicates how much they differed from the normal value [(raw TDD score − threshold)/threshold]. A negative value indicates a TDD score lower than the threshold value (i.e. a normal result) and a positive value indicate a TDD score higher than the threshold value. Shaded cells indicate TDD score above normal value. Lesions in bold are areas shown to be important for TDD in previous fMRI studies (Backlund Wasling *et al.*, 2008; Lundblad *et al.*, 2010, 2011).

BS: brain stem; CN: caudate nucleus; IC: insular cortex; L: left; LN: lentiform nucleus; R: right; S2: second somatosensory cortex; WM: white matter.

a Side difference above normal value.

### Brain lesions in relation to tactile direction discrimination

**Figure 4:**
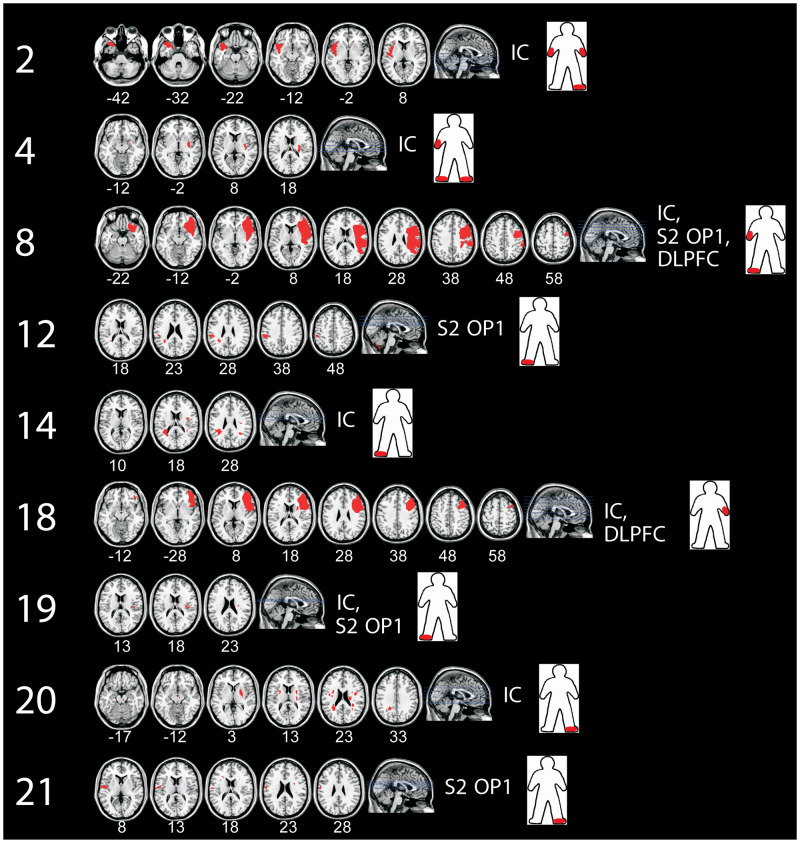
**Lesion maps for the nine patients with lesions affecting target areas.** These patients had lesions in S2 OP1, DLPFC, IC or in combinations thereof, i.e. in areas shown to be important for TDD in previous fMRI studies in healthy participants (Backlund Wasling *et al.*, 2008; Lundblad *et al.*, 2010, 2011). The red colour in the brain images indicates the lesions. The right side of the brain images corresponds to the right hemisphere. All of these patients had abnormal TDD. The red marks in the human figure, as seen from behind, indicate where abnormal TDD was observed. Numbers refer to patient number in Table 2.

**Figure 5:**
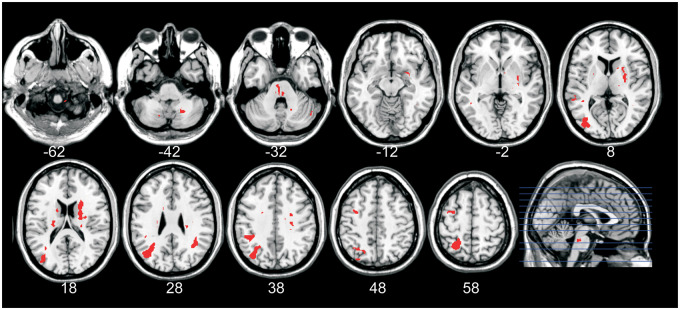
**Summary of lesions in the 12 patients with abnormal TDD and lesions outside target areas.** The target areas were S1, S2 OP1, IC and DLPFC (areas found to be important for TDD in previous fMRI studies in healthy participants; Backlund Wasling *et al.*, 2008; Lundblad *et al.*, 2010, 2011). The red colour in the brain images indicates the lesions. The right side of the brain images corresponds to the right hemisphere. Lesions shown here were localized to white matter, brainstem, cerebellum, thalamus, medulla oblongata, pons, occipital, frontal and parietal lobules [see Table 2 for more information (patient; 1, 3, 5–7, 9–11, 13, 15–17)].

The target brain areas identified as important for TDD in previous fMRI studies in healthy participants were S1, S2 OP1, DLPFC (BA9) and IC (anterior insular cortex and posterior IC) (Backlund Wasling *et al.*, 2008; Lundblad *et al.*, 2010, 2011) All nine patients with lesions affecting one or more of these target regions had impaired TDD (Fig. 4). Of the 19 patients with lesions in other areas, but not affecting any target regions, 12 of these had impaired TDD. As predicted, we found that patients with lesions affecting the fMRI-identified areas had a significantly higher chance of having impaired TDD compared to those with lesions in other areas [Fisher’s exact test: 9 of 9 patients with lesions in target areas had impaired TDD, 12 of 19 patients with lesion in other areas had impaired TDD; 95% CI odds ratio = (1.08∞) *P* = 0.042]. Although this is consistent with previous evidence regarding the target areas, the high rate of TDD impairment in patients with lesions in other areas is surprising. In these 12 patients, lesions were found in the brain stem, cerebellum and in the periventricular white matter (Fig. 5). One patient (no. 5) had lesions in the grey matter including the frontal and parietal lobe in Brodmann areas 6 and 7 and also in area V2 in the occipital lobe.

**Figure 6:**
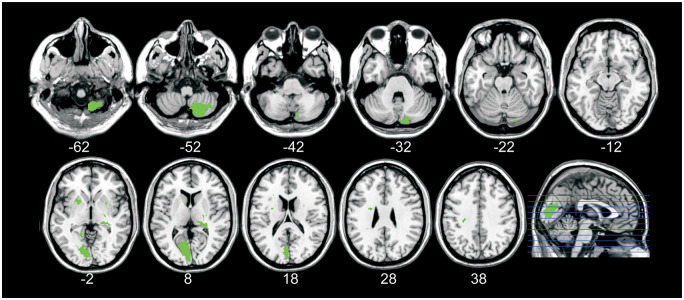
**Summary of the brain lesions in the seven patients with normal TDD.** The red colour in the brain images indicates the lesions. The right side of the brain images corresponds to the right hemisphere. The lesions were localized to white matter, brainstem, cerebellum, putamen, lentiform nucleus, caudate nucleus and the internal capsule [see Table 2 for more information (patient; 22–28)].

**Figure 7:**
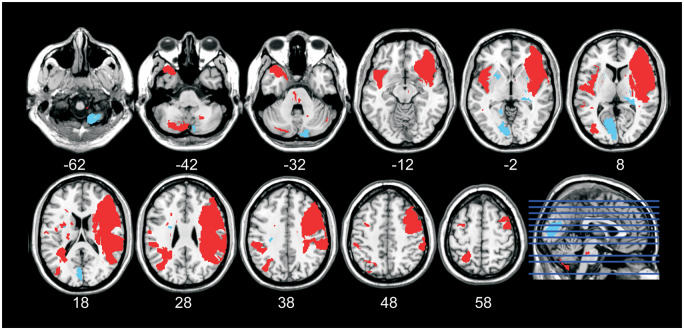
**Summary of the brain lesions in all patients.** The red colour in the brain images indicates the lesions from patients with abnormal TDD, and the blue colour indicates lesions from patients with normal TDD. The right side of the brain images corresponds to the right hemisphere.

Seven of the patients had normal TDD 3 months after the stroke (Table 2). Two of these patients had lesions restricted to the cerebellum. The remaining five patients with normal TDD had lesions in the brain stem and in the periventricular white matter, and one of those five patients had a lesion in the left occipital lobule (Fig. 6). Importantly, none of the patients had lesions in any of the target areas defined by fMRI to be important for TDD [S1, S2 OP1, DLPFC (BA9) and IC (anterior insular cortex and posterior IC)] (Backlund Wasling *et al.*, 2008; Lundblad *et al.*, 2010, 2011). Figure 7 contrasts the lesions from patients with impaired TDD with those with unimpaired TDD.

We tested direction discrimination on both the left and right sides of the body, allowing us to determine the laterality of impairment relative to lesion location. Of the 21 patients with TDD impairment, 11 had abnormal TDD contralateral to the lesion, four had an abnormal TDD ipsilateral to the lesion, and three had an abnormal TDD bilateral to the lesion (Table 2).

### Node-level symptom mapping

Patients with lesions in node 4 (Supplementary Fig. 2) had significantly worse left-side TDD scores (sum of hand and foot scores) at the 3-month follow-up compared to those without a lesion in node 4 [*t*(26) = −2.10, *P* = 0.023, one-tailed]. No significant difference was observed for right-side TDD scores [*t*(26) = 0.19, *P* = 0.573, one-tailed; Holm Bonferroni correction for these two tests produced a critical alpha of 0.025].

## Discussion

We found that TDD was impaired in the majority of a group of first-time sufferers of stroke, in both the acute testing and 3 months after the stroke. The likelihood of impairment was significantly greater for those participants with strokes in areas previously identified as important for TDD from fMRI studies in healthy individuals, including S2, IC and DLPFC (Backlund Wasling *et al.*, 2008; Lundblad *et al.*, 2010, 2011). Although the S1 is also important for tactile motion processing (Pei *et al.*, 2010; Planetta and Servos, 2012; Dépeault *et al.*, 2013), none of the patients in our study had a lesion in that area. A recently published study found a correlation in stroke patients between lesions in S1 and impairment on a variety of tactile sensory tasks, although they did not test tactile motion processing (Kessner *et al.*, 2019).

In the current study, we found lesions in the OP1 of the secondary somatosensory cortex (S2 region) in four patients, and all had impaired TDD, although none had a lesion exclusively in this area. There is consistent evidence that the S2 region is activated during tasks that require tactile motion processing (Burton *et al.*, 1999; Bodegård *et al.*, 2000; Downar *et al.*, 2000; Bremmer *et al.*, 2001; Olausson *et al.*, 2001, 2002; Beauchamp *et al.*, 2007; Backlund Wasling *et al.*, 2008; Lundblad *et al.*, 2010, 2011). However, the OP1 area most likely processes tactile input in general, rather than tactile motion specifically. Evidence for this is that it is activated in response to a variety of tactile stimuli (Burton *et al.*, 2008), and that lesions affecting the parietal operculum are associated with impaired somatosensory processing in tasks that do not require processing of motion cues (Preusser *et al.*, 2015; Meyer *et al.*, 2016; Lamp *et al.*, 2019). Furthermore, S2 OP1 activation appears unaffected by the performance level on the TDD test (Lundblad *et al.*, 2011), and was still activated during TDD testing in a patient with a spinal cord lesion who, although he reported sensing the tactile stimulus, was unable report movement direction (Lundblad *et al.*, 2010).

Lesions in the insula were found in seven of our patients and all of them had impaired TDD, including one (No. 2) without lesions elsewhere in the brain. Anterior IC is known to be involved in interoceptive awareness of factors of autonomic regulation but also in stimulus attention including attention to tactile stimuli (Albanese *et al.*, 2009; Craig, 2009; Lundblad *et al.*, 2010). The posterior IC is activated when a stimulus is moved over the skin (Lundblad *et al.*, 2011), and the activation correlates with sensory discriminative functions (Mazzola *et al.*, 2012, Kessner *et al.*, 2019). The IC also has strong functional connections with the S2 OP1 region (Wei and Bao, 2013). From the periphery, the IC receives input from myelinated afferents as well as from low-threshold unmyelinated mechanoreceptive (C-tactile) afferent fibres (Davidovic *et al.*, 2019), which are thought to play an important role in affective touch (McGlone *et al.*, 2014). Unlike myelinated afferents, C-tactiles are tuned to respond to slowly moving stimuli (Löken *et al.*, 2009), and their preferred speeds are similar to the optimal speed for TDD (Dreyer *et al.*, 1978; Essick and Whitsel, 1985). It thus seems possible that the C-tactile afferent insular pathway may play a role in TDD processing in addition to its role in affective touch (Marshall *et al.*, 2019). However, the task specificity of insular involvement with the TDD task is unclear. Lesions of the insular cortex lead to general somatosensory deficits affecting tasks not requiring motion processing (Preusser *et al.*, 2015; Meyer *et al.*, 2016).

We found two patients with lesions in the DLPFC, both with impaired TDD. However, both patients also had lesions in the insular region. Although this result does not allow for any strong conclusions, it is consistent with previous evidence that the DLPFC is important for decision-making in the somatosensory domain (Pleger *et al.*, 2006; Albanese *et al.*, 2009; Lundblad *et al.*, 2010, 2011; Adhikari *et al.*, 2014). One possible explanation is that DLPFC lesions resulted in pathological TDD by causing deficits in cognitive and attentional processing, which was clinically examined but not systematically tested in this study. However, one of the patients (No. 8) showed a strong contralateral impairment in TDD on both the hand and the foot, while the ipsilateral TDD performance was very good. The other patient (No. 18) had impaired TDD only on the right hand. The presence of good performance results on some of the tested sites for these two patients suggests that they were not severely impaired by cognitive or attentional factors (cf. exclusion criteria).

Importantly, our findings provide evidence that other areas not previously identified may also play a critical role and suggest that a wide set of brain regions is necessary for TDD. In addition to the areas previously identified in fMRI as being associated with TDD (S2, IC and DLPFC), we found impaired TDD in 12 of 19 patients with lesions in other areas. Although our sample did not permit us to isolate the contributions of specific regions, these additional areas included white matter (including capsula interna), brainstem, cerebellum, thalamus, medulla oblongata, pons and the frontal and parietal lobules. Our evidence is consistent with previous studies that indicate that the cerebellum, at least, may not be critical for tactile motion processing. We found that two patients (Nos 24 and 25) had lesions restricted to the cerebellum alone and had normal TDD values. Although sometimes activated in fMRI (Backlund Wasling *et al.*, 2008; Lundblad *et al.*, 2011), the cerebellum does not appear to be critical for normal TDD performance. The cerebellar fMRI activation may represent processing not related to the TDD but more generally to positioning of the stimulated limb (Proville *et al.*, 2014). In contrast, white matter tracts have been implicated in somatosensory deficits (Borstad *et al.*, 2012), and may be important for TDD.

In addition to the expected contralateral deficits, several patients had bilateral or ipsilateral TDD impairment at 3 months, and this was seen for hemispheric lesions of both left and right sides. This is perhaps not surprising given that verbal report of direction in a TDD task (as ours was) relies on an intact inter-hemispheric connection (Norrsell, 1973), and Backlund *et al.* (2005) found similar results. Furthermore, ipsilateral impairment in somatosensory function is evident in 20–30% of patients with unilateral brain lesions (Corkin *et al.*, 1973; Connell *et al.*, 2008). TDD bilaterally activates S2 and insula (Lamp *et al*., 2019; Lundblad *et al.*, 2011), and restricted experimental lesions alter cortical processing in widespread areas (Wahlbom *et al.*, 2019). A recent meta-analysis of functional neuroimaging studies of tactile processing of stimulation applied to the hand found bilateral activation in both S2 and the insula (Lamp *et al.*, 2019).

One limitation of our study is that although we recruited patients with no known history of stroke, identification of lesions was based on T2-weighted anatomical protocols and we cannot rule out that some of the lesions might be part of a cerebral microangiopathy or previous silent stroke lesion. We also observed what appears to be a floor effect with the TDD measurements of the hand in the healthy group. Using this version of the task means that we most likely underestimated the disturbance in the patients because it may have been easy enough that they could succeed even in the presence of some dysfunction. While a more difficult version of the task may have provided a better measure, the current version was sufficient to reveal dysfunction in the hand in 11 of the 28 patients 3 months after stroke. Another limitation is that the age range for stroke (27–82 years, mean 59 years) extends beyond the age range of the normative data (22–68 years, mean 49 years). This is somewhat mitigated by our approach of using thresholds adjusted for age, based on the normative data, instead of directly comparing the scores of the two groups. The threshold values were still extrapolated beyond the age range present in the normative data set, but this is reasonable, given that tactile sensory function continues to decline linearly with age beyond the age of our oldest patients (Gescheider *et al.* 1996; Stevens and Choo 1996; Olausson *et al.*, 1997).

We found that a high rate of abnormal TDD results in first time sufferers of stroke affecting a variety of brain areas. Our study reports that as many as 75% of first-time stroke patients have disturbed TDD, although we had a notable drop-out rate from the acute phase to the 3-month follow-up (from 44 to 28). This high rate of TDD disturbance might be because direction of tactile motion relies on processing in a large number of cortical regions, leaving it vulnerable to disturbance. Some evidence that TDD is reliant on a wider set of regions than other touch tasks comes from patients who have undergone hemispherectomy and lost the capacity for TDD but retained intact touch detection on their paretic body half (Backlund *et al.*, 2005). Furthermore, TDD testing has the highest sensitivity and specificity among neurological testing of patients with diabetic neuropathy, compared to nerve conduction velocity or cool sensitivity (Norrsell *et al.*, 2001), or to vibration detection (Löken *et al.*, 2010). Future studies with samples with more homogenous stroke lesion locations would be helpful in clarifying the role of these additional areas that we identified as potentially being important for tactile direction processing.

This hypothesis that tactile direction processing relies on a large number of brain regions suggests that TDD provides a sensitive clinical assessment tool for detecting disturbances in somatosensory processing. This is independent of the functional significance of TDD capacity, which remains unclear. Somatosensory function in general contributes to adequate grip force (Nowak *et al.*, 2003) and object manipulation (Hermsdörfer *et al.*, 2003), and tactile function specifically contributes to both proprioception (Edin, 1992) and postural control (Norrsell *et al.*, 2001; Backlund *et al.*, 2005). Deficits in somatosensory function relate to progress and outcome of the rehabilitation process after stroke (Carey *et al.*, 1993; Winward *et al.*, 1999) and can impact activities in daily living (Patel *et al.*, 2000; Birznieks *et al.*, 2012), as well as independence and recovery (Tyson *et al.*, 2008). It is still unknown to what extent TDD is required for these functional outcomes, or if it is simply an indicator of neurological disturbance.

## Conclusions

We have studied the capacity to discriminate the direction of touch that moves across the skin in patients with first-time stroke and shown that a large number have impaired direction discrimination, both when tested within 2 weeks from the stroke (66%), and 3 months later (75%). Abnormal direction discrimination was associated with lesions in areas that were previously identified to be active during a TDD task (S2, IC and DLPFC) using fMRI with healthy participants. We now confirm an important role for these regions in tactile direction processing. We also found abnormal direction discrimination in 12 of 19 patients with lesions in other brain regions including white matter, brainstem, cerebellum, thalamus, medulla oblongata, pons, frontal and parietal lobules, suggesting that a larger number of areas than previously thought may be critical for processing the direction of tactile motion.

## Supplementary material

Supplementary material is available at *Brain Communications* online.

## Supplementary Material

fcaa088_Supplementary_Data

## Abbreviations

DLPFC: = dorsolateral prefrontal cortex
fMRI: = functional magnetic resonance imaging
IC: = insular cortex
S1: = primary somatosensory cortex
S2 OP1: = opercular parietal area 1 of the secondary somatosensory cortex
TDD: = tactile direction discrimination
