# Supplementary material for: Tactile direction discrimination in humans after stroke
Source: Brain Commun. 2020 Jun 30;2(2):fcaa088. doi: 10.1093/braincomms/fcaa088 (PMC7472910; doi:10.1093/braincomms/fcaa088)
Supplement: fcaa088_Supplementary_Data [file fcaa088_supplementary_data.docx]

**Supplementary Figure 1** Node 4 of 43 distinct, contiguous sub-regions from the seven-network, liberal parcellation from Yeo *et al.*, (2011). This was the only node that met criteria for statistical analysis for the ‘Node-level symptom mapping’ technique.

**
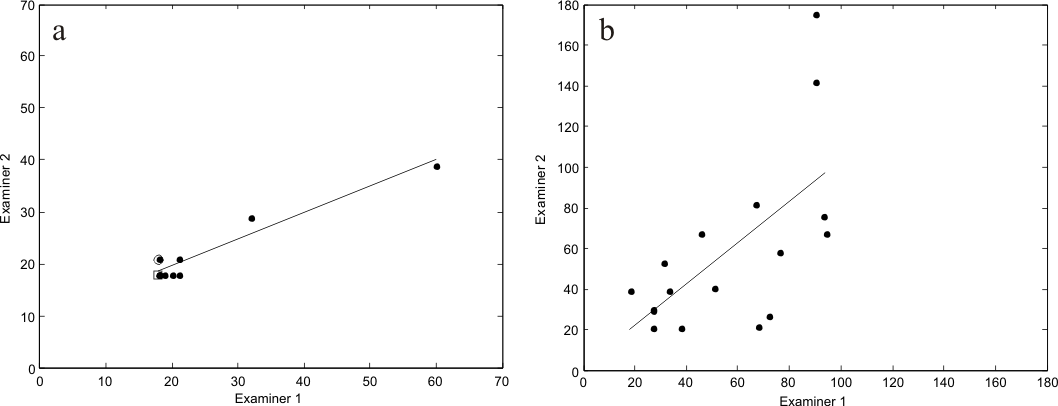
**

**Supplementary Figure 2** Correlation between examiner 1 on the x-axis and examiner 2 on the y-axis for TDD **a** on the left hand (R^2^=0.9276) and **b** on the left foot (R^2^=0.3916). The open circle in **a** indicate two subjects with the same value and the open square indicate ten subjects with the same value (lowest possible score).
